# Effect of castration timing and weaning strategy on the taxonomic and functional profile of ruminal bacteria and archaea of beef calves

**DOI:** 10.1186/s42523-023-00284-2

**Published:** 2023-12-01

**Authors:** Gerardo R. Diaz, Tara N. Gaire, Peter Ferm, Lacey Case, Luciano S. Caixeta, Timothy J. Goldsmith, Joe Armstrong, Noelle R. Noyes

**Affiliations:** 1 Department of Veterinary Population Medicine, College of Veterinary Medicine, University of Minnesota, St. Paul, MN 55108 USA; 2 North Central Research and Outreach Center, Department of Animal Science, University of Minnesota, St. Paul, MN 55108 USA; 3 Agricultural and Natural Resource Systems, University of Minnesota Extension, University of Minnesota, St. Paul, MN 55108 USA

**Keywords:** Cattle, Early life, Microbiome, Methanogens, Resistome, Fence line, Truck, Gastrointestinal, Shotgun

## Abstract

**Background:**

Beef cattle experience several management challenges across their lifecycle. Castration and weaning, two major interventions in the early life of beef cattle, can have a substantial impact on animal performance. Despite the key role of the rumen microbiome on productive traits of beef cattle, the effect of castration timing and weaning strategy on this microbial community has not been formally described. We assessed the effect of four castration time windows (at birth, turnout, pre-weaning and weaning) and two weaning strategies (fence-line and truck transportation) on the rumen microbiome in a randomized controlled study with 32 male calves across 3 collection days (i.e., time points). Ruminal fluid samples were submitted to shotgun metagenomic sequencing and changes in the taxonomic (microbiota) and functional profile (metagenome) of the rumen microbiome were described.

**Results:**

Using a comprehensive yet stringent taxonomic classification approach, we identified 10,238 unique taxa classified under 40 bacterial and 7 archaeal phyla across all samples. Castration timing had a limited long-term impact on the rumen microbiota and was not associated with changes in alpha and beta diversity. The interaction of collection day and weaning strategy was associated with changes in the rumen microbiota, which experienced a significant decrease in alpha diversity and shifts in beta diversity within 48 h post-weaning, especially in calves abruptly weaned by truck transportation. Calves weaned using a fence-line weaning strategy had lower relative abundance of *Bacteroides, Lachnospira, Fibrobacter* and *Ruminococcus* genera compared to calves weaned by truck transportation. Some genes involved in the hydrogenotrophic methanogenesis pathway (*fwdB* and *fwdF*) had higher relative abundance in fence-line-weaned calves post-weaning. The antimicrobial resistance gene *tetW* consistently represented more than 50% of the resistome across time, weaning and castration groups, without significant changes in relative abundance.

**Conclusions:**

Within the context of this study, castration timing had limited long-term effects on the rumen microbiota, while weaning strategy had short-term effects on the rumen microbiota and methane-associated metagenome, but not on the rumen resistome.

**Supplementary Information:**

The online version contains supplementary material available at 10.1186/s42523-023-00284-2.

## Background

The microbial community inhabiting the rumen of cattle, termed the rumen microbiome, can generate up to 70% of animal energy needs from inedible feedstuffs via fermentation processes [1]. These processes also result in the generation of greenhouse gases (e.g., methane), which have been estimated to contribute up to 40% of all livestock emissions [2]. Multiple microorganisms of the rumen microbiome, made up of bacteria, fungi, archaea, protozoa and viruses [3], as well as their associated genes (i.e., metagenome), have been associated with relevant host traits, such as feed efficiency [4–7], methane emissions [8–11] and meat quality [12]. The bacterial and archaeal communities of the rumen microbiome have been shown to exert life-long influence on not only their host, but also that host’s offspring [13, 14]. For example, the initial colonization of microbes in the neonatal calf rumen is a significant predictor of rumen microbiome composition later in life, i.e., once the rumen has developed [15], and a small microbial core [14] and set of genes [12] of the rumen microbiome has been identified as heritable. In light of current evidence, manipulation of the rumen microbiome may be an effective strategy to improve many aspects of beef production, much like leveraging cattle genetics has led to myriad impacts across the beef production system. However, we still lack longitudinal studies to understand how external factors impact the establishment, composition and function of the rumen microbiome throughout the beef cattle life cycle [16, 17].

A typical beef cattle lifecycle can involve moving animals through several stages, including cow-calf, backgrounding, stocker, and feedlot [18]. As calves move through these stages, they experience different management practices that can include various physical interventions (e.g., castration, weaning, dehorning, vaccination); changes in diet, environment and herd; as well as transport [19]. Some of these changes happen suddenly and represent stressors that can affect a calf’s metabolism, immune system, health and performance, and microbiome [20]. Within the cow-calf stage, castration and weaning are two management events that can be stressful due to numerous physiological, environmental and management changes. However, these two events also represent opportunities for cow-calf producers to deploy practical management interventions. Such opportunities are fairly limited within cow-calf production because of its extensive nature and corresponding lack of intensive calf management.

Specific evidence shows that choice of strategy for castration [21], weaning [22], dehorning [23] and high-energy diet supplementation [24] can directly impact welfare, health and performance of beef cattle. Similarly, a growing body of evidence suggests that the rumen microbiome of beef cattle is also impacted by these management practices, including diet changes [25–29] and stress factors [30]. Despite the importance of early-life events for both long-term beef cattle performance [31] and rumen microbiome assembly and establishment [15], little is known about the effect of management practices on the beef calf rumen microbiome, especially in comparison to the body of literature that pertains to dairy calves [32–35]. Furthermore, most previous studies related to the rumen microbiome of beef calves are based on 16 S rRNA gene sequencing [17, 26, 36], which precludes characterization of the putative function of ruminal microbes. The genetic capacity of the rumen microbiome has been shown to play an important role in antimicrobial resistance [37], methane emissions [8] and productivity [6] of adult cattle, but has been largely under-explored in young animals. By focusing on gene-level information (metagenome), the present study substantially contributes to understanding the potential function of the rumen microbiome during the early life of beef cattle. Specifically, we hypothesized that castration timing and weaning strategy influence the diversity, taxonomy and potential functional profile of the rumen microbiome of beef calves. To test this hypothesis, we conducted a longitudinal randomized controlled study with 32 male calves, aimed to evaluate the impact of 4 castration time windows (at birth, turnout, pre-weaning and weaning) and 2 weaning strategies (fence-line and truck transportation) on the rumen microbiota and the metagenome associated with 2 microbe-driven processes: antimicrobial resistance (AMR) and methane emissions.

## Results

### Study population description

**Fig. 1:**
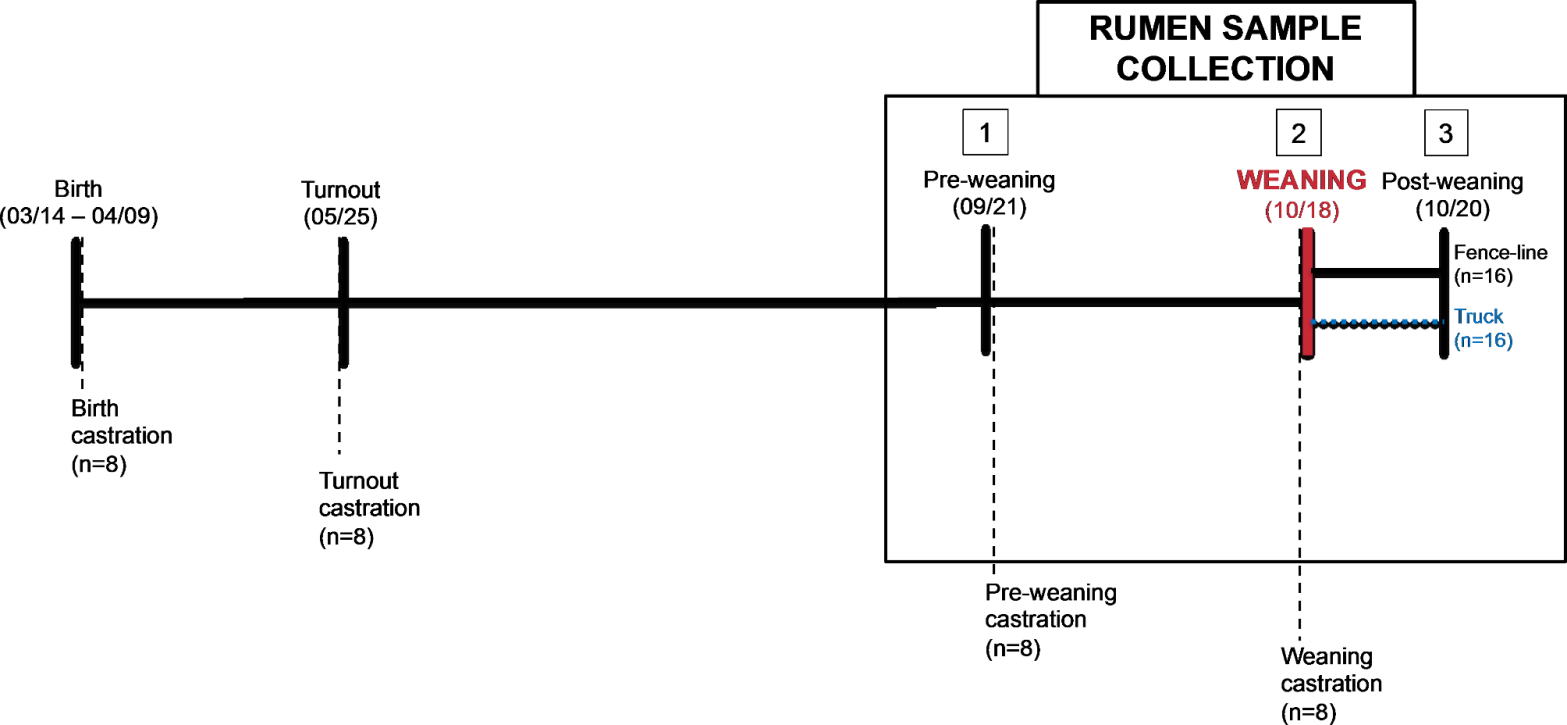
Study timeline. Study calves were born during a 26-day calving window (March 14 – April 9), and then were turned out to pasture on May 25. Study calves were sampled 3 times: (**1**) at pre-weaning processing on September 21; (**2**) at weaning on October 18; (**3**) at post-weaning on October 20. Castration interventions are depicted by vertical dashed lines, producing 4 castration timing groups. Weaning intervention is depicted by a vertical red line, producing 2 weaning strategy groups

At birth, 32 male beef calves were enrolled into the study and randomly allocated to 4 castration timing groups (birth, turnout, pre-weaning and weaning) and 2 different weaning strategies (fence-line and truck transportation) in a 4 × 2 factorial design. The rumen fluid of each calf was sampled at 3 time points (Fig. 1), for a total of 95 samples (one calf assigned to turnout castration and fence-line weaning was dropped from the study due to health issues that occurred at weaning and resulted in euthanasia). Calf weights at birth and post-weaning were not statistically different between treatment groups at any time point (Table 1 and Sup Fig. 1A). Average Daily Gain (ADG) and calf age were not significantly different between castration groups (Table 1 and Sup Fig. 1B). However, despite formal randomization, there was a significant difference in ADG and age of calves assigned to the 2 weaning groups (Kruskal-Wallis Test, *P* < 0.05). Calves weaned by fence-line had on average 0.11 kg higher ADG and were on average 6 days younger than truck-weaned calves without adjusting by any covariate (Table 1). Random allocation of calves to weaning groups at birth occurred over a 26-day calving window, while the weaning process occurred for all calves on a single date. Thus, these results were not unexpected, and we adjusted for them by adding weight and age as covariables in our statistical models to account for potential confounding between weaning groups and microbiome outcomes.

**Table 1:** Summary of calf ages and weights, by castration timing and weaning groups (mean ± SE)

Variable	Castration				*P* values*	Weaning		*P* values*
	Birth	Turnout	Pre-weaning	Weaning		Fence-line	Truck	
Weight at birth (kg)	38.21 ± 1.99	37.06 ± 1.74	38.1 ± 1.66	37.64 ± 1.07	0.9	38.52 ± 0.95	37.07 ± 1.24	0.2
Weight at Post-weaning (kg)	263.83 ± 7.67	267.44 ± 8.17	267.18 ± 11.08	275.06 ± 8.95	0.8	276.52 ± 6.89	260.8 ± 5.05	0.1
Average daily gain (kg)	1.06 ± 0.04	1.09 ± 0.04	1.1 ± 0.05	1.14 ± 0.05	0.6	1.15 ^a^ ± 0.03	1.04 ^b^ ± 0.02	0.02
Age at Post-weaning (days)	213 ± 2.56	212 ± 1.56	209 ± 3.32	209 ± 3.71	0.8	208 ^c^ ±2.17	214 ^d^ ±1.63	0.01

*Kruskal-Wallis test

^a,b,c,d^Values with different superscript letters within the same row denote statistically significant differences

### Rumen microbiota: diversity and differential abundance over time

**Fig. 2:**
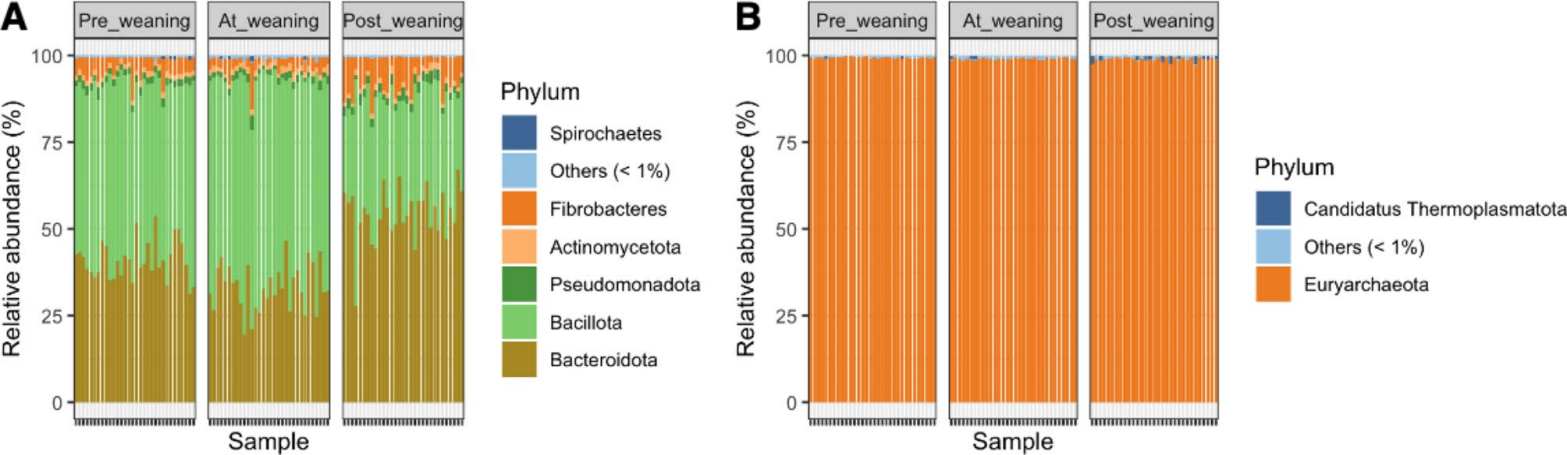
Relative abundance plots of phylum-level microbiota composition for (**A**) Bacteria and (**B**) Archaea grouped by collection day (Pre_weaning, At_weaning and Post_weaning). Phyla with < 1% relative abundance are grouped as “Others”. Each bar represents one sample

After shotgun metagenomic sequencing, a total of 10,894 OTUs (operational taxonomic units) were identified across all samples by Kraken 2 (confidence score = 0.1) [38] using a rumen-specific reference database. We focused our downstream analysis on the bacterial and archaeal domains of the rumen microbiota, filtering out virus, plasmids, human, UniVec_core, protozoa, and fungi reads, resulting in 10,238 remaining OTUs. Within the bacterial domain (98.6% of total classified reads), 40 unique phyla, 93 classes, 219 orders, 519 families, 1909 genera and 8674 species were identified across all rumen fluid samples (Sup. Table 1). Within the archaeal domain (1.4% of total classified reads), 7 unique phyla, 18 classes, 31 orders, 48 families, 140 genera and 351 species were identified (Sup. Table 1). Overall, the most abundant bacterial phyla across all ruminal fluid samples were *Bacillota* (48.1% ± 13%, mean ± SD), *Bacteroidota* (42.7% ± 11%), *Fibrobacteres* (5.16% ± 3.48), *Pseudomonadota* (2.05% ± 0.62), *Actinomycetota* (1.57% ± 0.36) *and Spirochaetes* (1.28% ± 0.24) (Fig. 2).

The 10 most abundant bacterial genera across all ruminal fluid samples were *Prevotella* sp. (40.5% ± 10.9, mean ± SD), *Butyrivibrio* sp. (16.4% ± 7.72), *Selenomonas* sp. (5.50% ± 2.64), *Fibrobacter* sp. (5.46% ± 3.67), *Oribacterium* sp. (3.9% ± 1.31), *Succiniclasticum* sp. (3.73% ± 2), *Pseudobutyrivibrio* sp. (3.65% ± 1.73), *Ruminococcus* sp. (2.94% ± 1.28), *Eubacterium* sp. (1.8% ± 0.96), and *Sarcina* sp. (1.82% ± 0.78). Within the archaeal domain, the 5 most abundant genera were *Methanobrevibacter* sp. (83.8% ± 7.03), *Methanosphaera* sp. (14.7% ± 4.04), *Methanomicrobium* sp. (2.72% ± 2.49), *Methanosarcina* sp. (1.55 ± 0.1) and *Candidatus Methanoplasma* sp. (1.45% ± 0.39) (Sup. Figure 3).

**Fig. 3:**
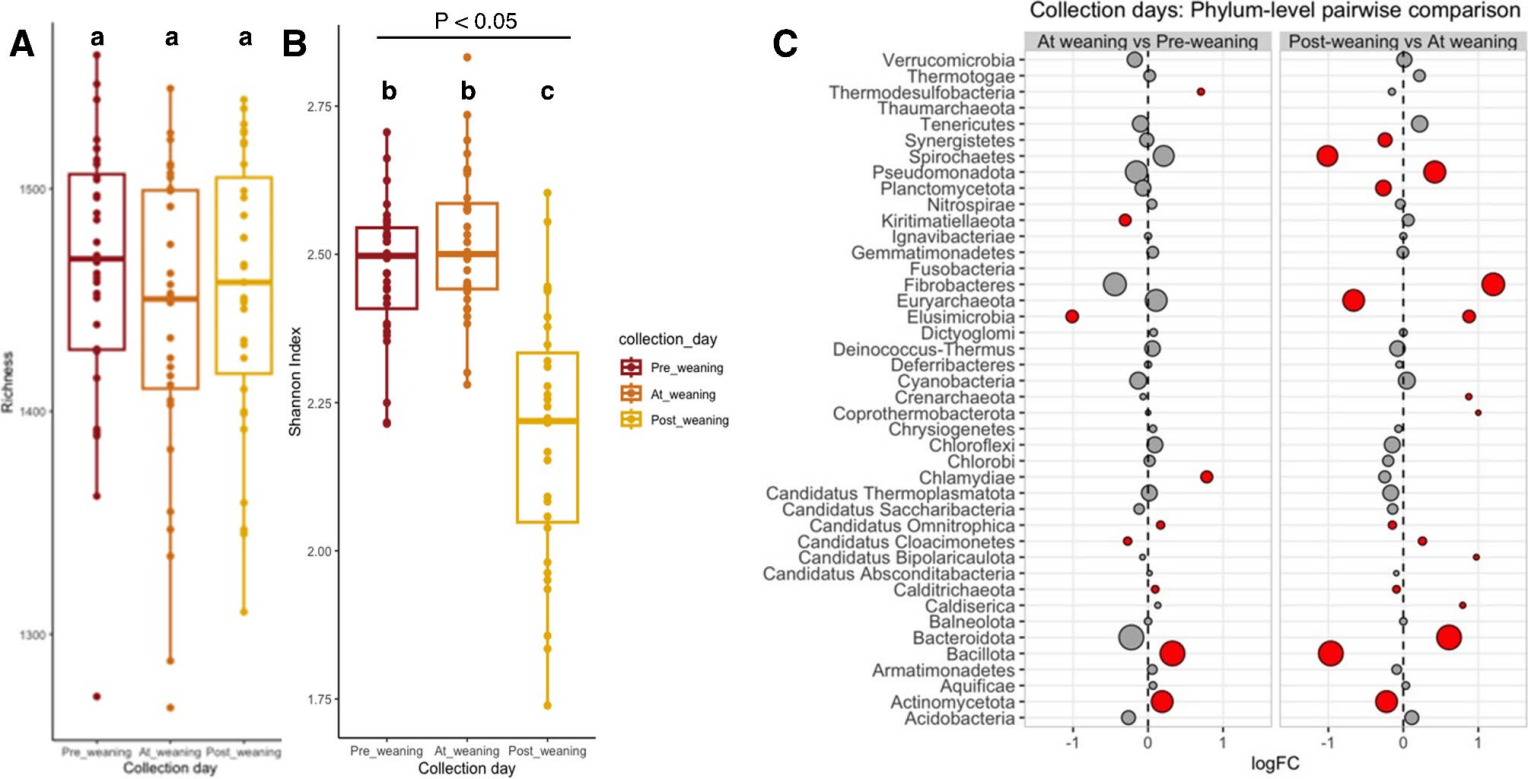
Rumen microbiota, comparisons over time. Box plots of (**A**) richness and (**B**) Shannon’s index, at the genus level, grouped by collection day. Boxes represent the 25th to 75th percentile; horizontal line represents the median; and whiskers indicate 1.5× the interquartile range (IQR), *P* values from Type-III ANOVA, collection days with different superscript letters were significantly different. (**C**) Differential abundance of phylum-level counts between collection days, expressed as log_2_ fold change (LogFC). Statistically significant logFC values (adjusted *P* < 0.05) are depicted in red, and non-significant in grey. Circle diameter is proportional to the average abundance of each phylum across all samples

**Fig. 4:**
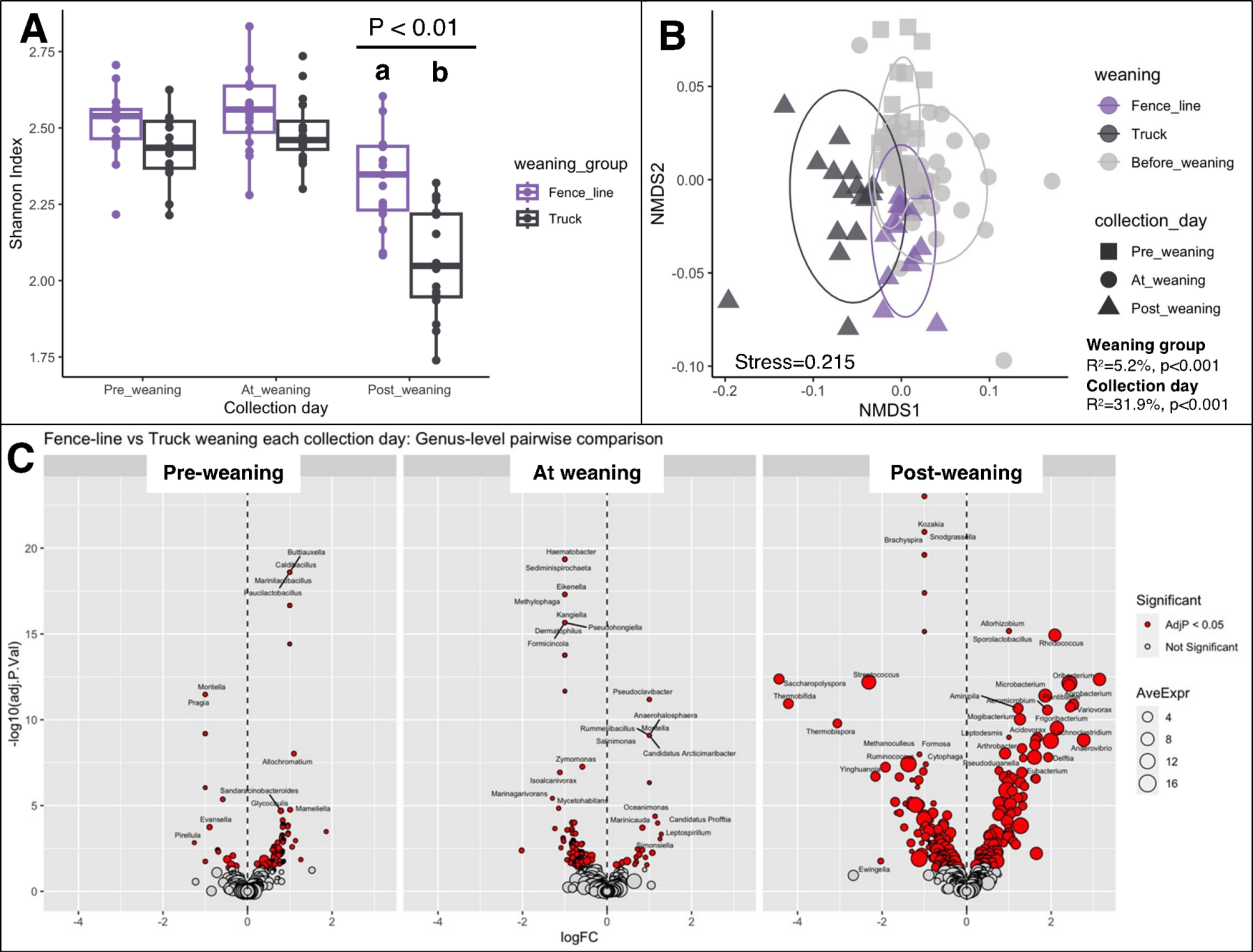
Rumen microbiota, differences by weaning strategy. (**A**) Box plots of Shannon’s Index at the genus level, stratified by weaning strategy across collection days. Boxes represent the 25th to 75th percentile; horizontal line represents the median; and whiskers indicate 1.5× the interquartile range (IQR), *P* values from Type-III ANOVA, weaning groups with different superscript letters were significantly different. (**B**) Non-metric multidimensional scaling (NMDS) ordination plots based on Bray–Curtis distances at the genus level colored by weaning strategy. *P* value and R^2^ values from PERMANOVA testing. (**C**) Differential abundance of microbial genera between fence-line and truck weaned calves at different collection days, expressed as log2 fold change (LogFC). Statistically significant logFC (adjusted *P* < 0.05) are depicted in red and non-significant in grey. Circle diameter is proportional to the average abundance of each phylum across all samples

Using a linear mixed effects model to assess the association between genus-level alpha diversity indices (richness and Shannon’s index) and collection day, castration timing and weaning strategy, we found that collection day was significantly associated *(ANOVA-III, P <* 0.001) with Shannon’s index, but not with richness (Fig. 3A). Specifically, rumen samples collected at post-weaning had significantly lower Shannon’s diversity (adjusted mean ± SE, 2.18 ± 0.03) than before weaning (2.50 ± 0.03) (Fig. 3B). In addition, the permutational multivariate analysis of variance (PERMANOVA) test for beta diversity revealed that a significant amount of rumen microbiota variability between samples was partitioned to collection day (R^2^ = 31.9%, *P* < 0.001) (Fig. 4B).

To investigate which specific members of the rumen microbiota were driving these differences in diversity, we used a multivariate zero-inflated Gaussian mixture model to measure the differential abundance (log_2_ fold change) of rumen phyla between collection days. We observed numerous differentially abundant phyla when comparing samples collected at weaning and post-weaning. Specifically, eight phyla were in higher relative abundance at post-weaning, while seven were in lower relative abundance. Considering only phyla with high average relative abundance across all samples, *Spirochaetes, Euryarchaeota* and *Bacillota* were in lower relative abundance post-weaning compared to at-weaning (log_2_ fold change values: -1.01, -0.66, -0.97, respectively; BH-adj. *P* < 0.05), while *Fibrobacteres* and *Bacteridota* were in higher relative abundance (log_2_ fold change values: 1.2 and 0.61; BH-adj. *P* < 0.05) (Fig. 3C).

### Rumen microbiota: diversity and differential abundance between weaning strategy and castration timing

Not only collection day but also its interaction term with weaning strategy was significantly associated with alpha diversity of the rumen microbiota (*ANOVA-III, P < 0.001*). Although fence-line and truck weaned calves had small diversity differences between pre-weaning and at-weaning collection days, a statistically significant difference was only detected at post-weaning. Specifically, rumen samples collected from truck-weaned calves after weaning had significantly lower diversity compared to the post-weaning samples from fence-line weaned calves (β = -0.18, 95% CI = -0.31, -0.05) (Fig. 4A). Weaning strategy was associated with 5.2% (PERMANOVA, *P* < 0.01) of the overall variability in beta diversity across all of the rumen samples (Fig. 4B). However, when only post-weaning samples were analyzed, weaning strategy was associated with 32.5% (PERMANOVA, *P* < 0.01) of the between-sample variability.

Differential abundance testing by weaning strategy stratified by collection day showed that the majority of differences between weaning groups were observed at the post-weaning time point. Specifically, 564 genera identified in the post-weaning samples were significantly differentially abundant between fence-line and truck-weaned calves, compared to 266 and 239 genera at the pre-weaning and weaning collection days, respectively (BH-adj *P* < 0.05) (Fig. 4C). Considering only the most abundant genera across all samples, we identified *Bacteroides*, *Lachnospira*, *Petrimonas, Micromonospora, Fibrobacter, Sarcina, Streptococcus* and *Ruminococcus* genera as having significantly lower relative abundance (log_2_ fold change < -1; BH-adj. *P* < 0.05) in fence-line-weaned calves compared to truck-weaned calves post-weaning (i.e., fence-line-weaned calves had less than 0.5 times the relative abundance of truck-weaned calves); while the *Rhodococcus, Agrobacterium, Anaerovibrio, Oribacterium, Plantibacter, Variovoraxgenera* and *Lachnoclostridium* genera were in higher abundance (log_2_ fold change > 2; BH-adj. *P* < 0.05) (i.e., fence-line-weaned calves had more than 4 times the relative abundance of truck-weaned calves) (Sup. Table 2).

**Fig. 5:**
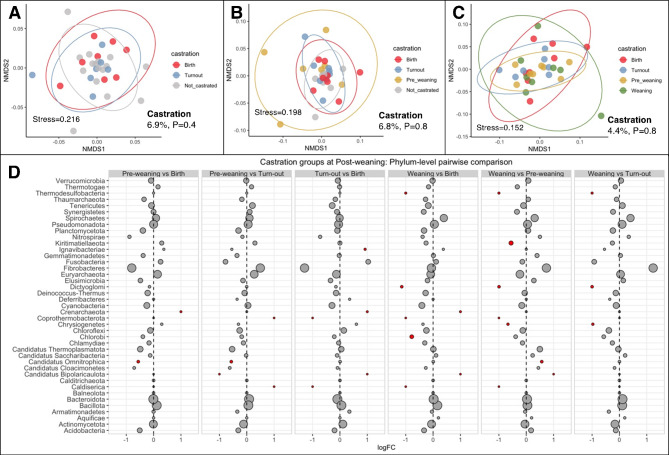
Rumen microbiota, differences by castration timing. Non-metric multidimensional scaling (NMDS) ordination plots based on Bray–Curtis distances at the genus level for (**A**) Pre-weaning, (**B**) At weaning, and (**C**) Post-weaning collection days, colored by castration timing group. *P* value and R^2^ values from PERMANOVA testing (**D**) Differential abundance of microbial phyla between castration timing groups at post-weaning day expressed as log2 fold change (LogFC). Statically significant logFC (adjusted *P* < 0.05) are depicted in red and non-significant in grey. Circle diameter is proportional to the average abundance of each phylum across all samples

Unlike weaning strategy, castration timing was not significantly associated with differences in Shannon’s index at any collection day (pre-weaning *P* = 0.4, weaning *P* = 0.8 and post-weaning *P* = 0.8). When beta diversity was assessed separately for each collection day, the variation partitioned to castration timing was small and not statistically significant (Fig. 5A - C). Furthermore, differential abundance testing revealed limited statistically significant differences in relative abundance of any phyla when comparing castration timing groups at weaning (Sup. Figure 4) and post-weaning (Fig. 5D).

### Rumen metagenome: methane-associated microbes and genes, by weaning strategy

We assessed the effect of weaning strategy specifically on the methane-associated microbial community and genes, using a dedicated database and bioinformatic tool [39]. “Methane-associated” was defined as bacteria or genes known to be involved in methane cycling pathways, either to produce or to consume (i.e., oxidate) methane. The relative abundance of these microbes across time and between weaning strategies was highly heterogeneous (Sup Fig. 5A). As with the rumen microbiota, the association between methane-associated gene diversity and both weaning strategy and time were statistically significant. Shannon’s index for methane-associated genes was significantly lower in truck-weaned calves (adjusted means ± SE, 3.96 ± 0.01) compared to fence-line-weaned calves (4.03 ± 0.01) at post-weaning (*P* < 0.01) (Sup Fig. 5B). Likewise, a large proportion of the variation in the composition of methane-associated genes was attributed to weaning strategy (R^2^ = 8.1%, *P* < 0.01) and collection day (R^2^ = 24.3%, *P* < 0.01) (Sup. Figure 5C).

**Fig. 6:**
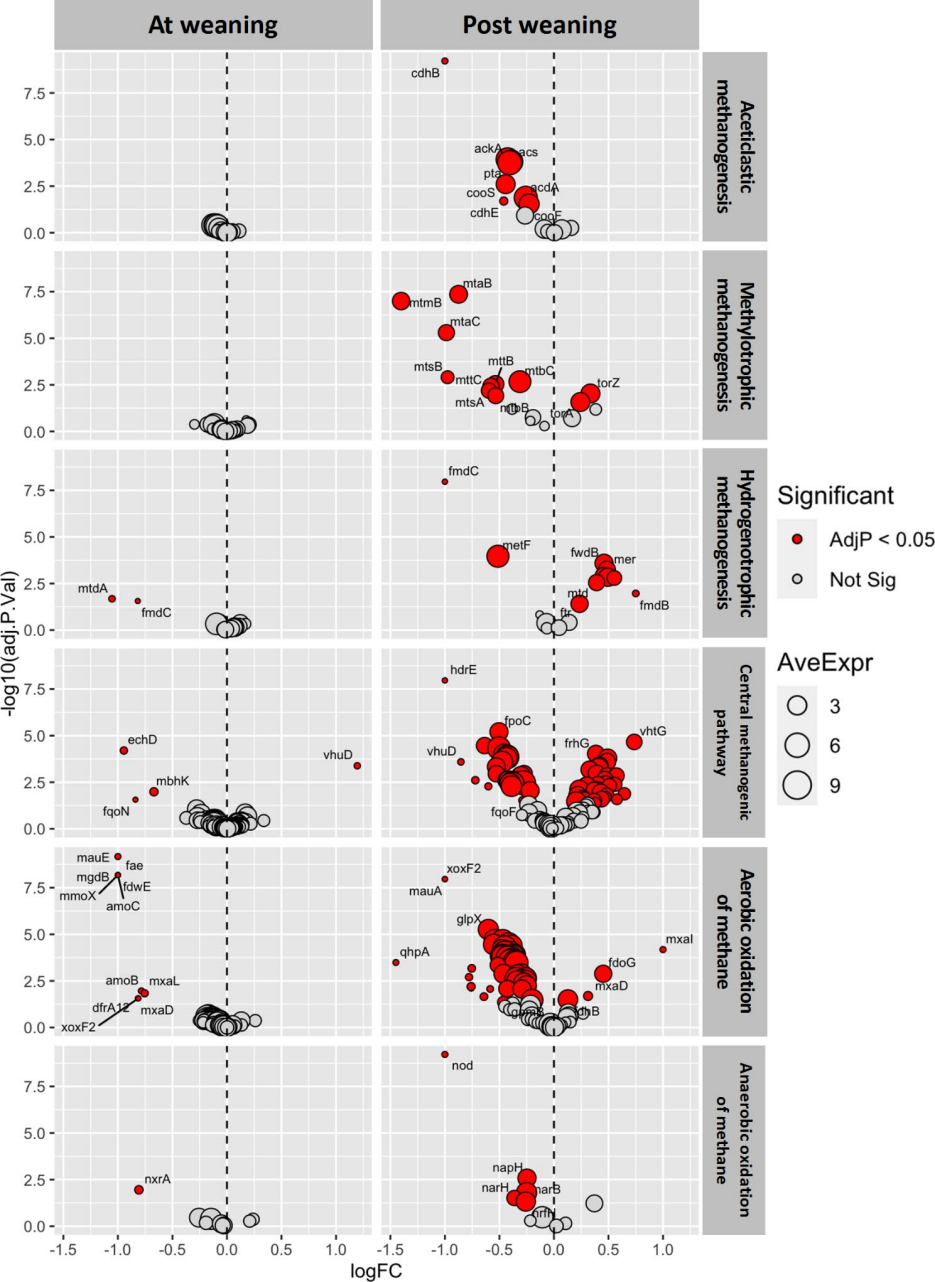
Differential abundance of methane-associated genes between fence-line and truck weaned calves at weaning and post weaning, stratified by methane cycling pathway and expressed as log2 fold change (LogFC). Statistically significant logFC (Benjamin-Hochberg adjusted *P* < 0.05) are depicted in red and non-significant in grey. Circle diameter is proportional to the average abundance of each gene across all samples

A total of 1139 methane-associated genera and 252 methane-associated genes were identified in post-weaning samples, and 64 and 98 of these, respectively, had significantly lower relative abundance in fence-line-weaned calves, while 45 genera and 45 genes had significantly higher relative abundance, when compared to truck-weaned calves (BH-adj. *P* < 0.05). For the differential abundance testing of methane-associated genes, we compared fence-line-weaned vs. truck-weaned calves at weaning and post-weaning and stratified our analysis by the 6 methane cycling pathways (i.e., acetoclastic methanogenesis, methylotrophic methanogenesis, hydrogenotrophic methanogenesis, central methanogenesis pathway, aerobic and anaerobic oxidation of methane). At weaning, minimal but significant differences in gene relative abundances were observed between fence-line and truck-weaned calves in the hydrogenotrophic methanogenesis (2 genes were lower in fence-weaned calves), central methanogenesis (1 gene was higher and 3 lower), aerobic (11 genes were lower) and anaerobic methane oxidation (1 gene was lower) pathways (Fig. 6). Post-weaning, more significant differences in gene abundance were observed. In the acetoclastic methanogenesis pathway 8 genes had lower relative abundance in fence-weaned versus truck-weaned calves; in the methylotrophic methanogenesis pathway 2 genes had higher and 9 lower; in the hydrogenotrophic methanogenesis pathway 8 genes had higher and 2 lower; in the central methanogenesis pathway 31 genes had higher and 27 lower; in the aerobic oxidation pathway 4 genes had higher and 47 lower; and in the anaerobic oxidation pathway 5 genes had lower relative abundance in fence- versus truck-weaned calves (Fig. 6). Within the most abundant methane-associated genera and genes across all samples, the archaeal genus *Candidatus Methanomethylophilus*, and some genes in the methylotrophic methanogenesis pathway (*mtmB* and *mtaB*) had significantly lower relative abundance in fence-line-weaned calves compared to truck-weaned calves; while the archaeal genus *Methanobrevibacter* and some genes in the hydrogenotrophic methanogenesis pathway (*fwdB* and *fwdF*) had significantly higher relative abundance (Sup. Table 3).

### Rumen metagenome: rumen resistome analysis

**Fig. 7:**
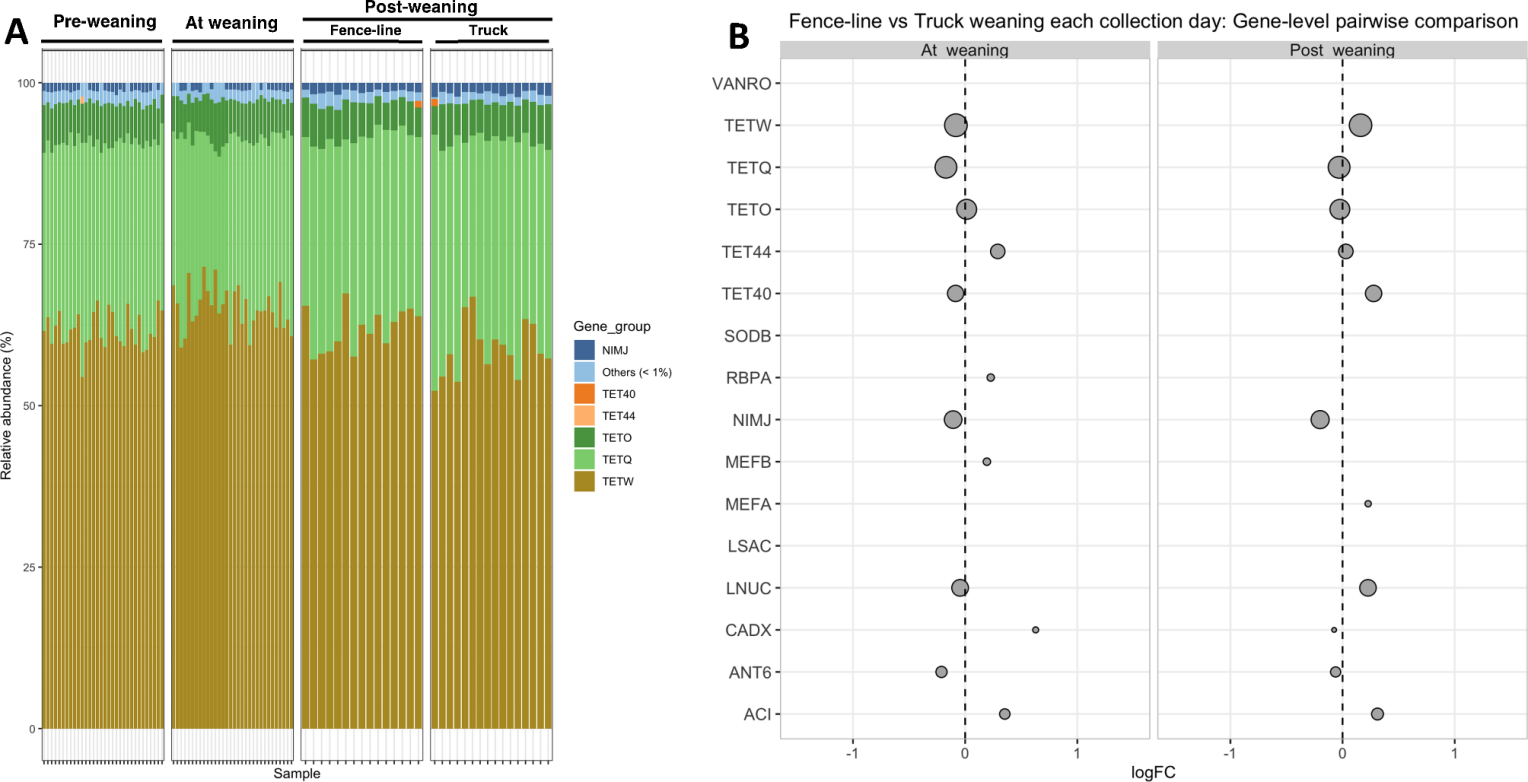
Rumen resistome by weaning strategy across collection days. (**A**) Relative abundance plot of antimicrobial resistance genes (ARGs) at the ARG group level, grouped by collection day and weaning strategy. ARGs with < 1% abundance are grouped as “Others”. Each bar corresponds to an individual sample. (**B**) Differential abundance of ARG groups between fence-line and truck weaned calves, at weaning and post-weaning days, expressed as log2 fold change (LogFC). Statistically significant logFC (adjusted *P* < 0.05) are depicted in red and non-significant in grey. Circle diameter is proportional to the average abundance of each ARG group across all samples

We assessed the Antimicrobial Resistance Genes (ARGs) in the rumen metagenome using AMR + + v2 [40]. Across the 95 samples, we found 111 ARGs distributed across 24 antibiotic classes and 49 mechanisms of resistance. The composition of ARGs within the rumen (i.e., the rumen resistome) did not change significantly over time nor did it differ significantly between weaning groups. More than 90% of the resistome at the gene-group level was composed of tetracycline resistance genes, namely *Tet40, Tet44, TetO, TetQ* and *TetW*, with the latter comprising more than half of the total resistome content (Fig. 7A). The homogeneous composition of tetracycline resistance genes was reflected in our analysis of both alpha and beta diversity, neither of which demonstrated significant associations with weaning strategy (data not shown). In addition, differential abundance testing revealed limited statistically significant differences between weaning groups at weaning and post-weaning collection days (Fig. 7B).

### Low classification rate but high taxonomic resolution of the rumen microbiome with Kraken 2

Shotgun metagenomic sequencing generated 5.1 × 10^9^ paired-end (PE) sequencing reads across all 95 samples (mean 55.8 × 10^6^ PE reads per sample, range 30.5–75.8 × 10^6^) with an overall mean quality score of 35.1. The initial standard protocol for taxonomic profiling of the rumen microbiome (see methods) classified a low proportion of total sequencing reads (Sup. Table 4). Based on a previous report [41], we customized our bioinformatic workflow for analyzing the metagenomic rumen sequence data, including removal of reads aligning to *Bos taurus* and dietary plant genomes (when available), followed by taxonomic classification using a customized database that included rumen-specific bacteria and archaea (see methods for details). After trimming low quality sequencing reads and removing host and dietary plant genome sequences, an average of 38.3 × 10^6^ PE reads per sample remained (70.7% of raw reads across all samples). The trimmed non-host reads were classified using Kraken 2 (confidence score = 0.1) [38] with the rumen-specific database, resulting in an average of 1.6 × 10^6^ PE classified reads per sample (4.2% of trimmed non-host reads across all samples). The addition of plant genomes to the “host removal” process increased the removal of non-microbiome-related reads, i.e., from 22.7% using only the *Bos taurus* genome to 29.3% using *Bos taurus* and dietary plant genomes. Additionally, the use of a rumen-customized microbial database increased the proportion of non-host reads classified by Kraken 2 (confidence score = 0.1), i.e., from 3.1% using the standard database to 4.2% using the rumen-specific database (Sup. Table 4). Overall, with our customized workflow, 96.2% of Kraken-classified reads were resolved to the phylum level, while 75.7% were resolved to the species level (Sup. Table 5).

Two positive controls (i.e., mock communities) sequenced alongside the rumen samples yielded 51.6 × 10^6^ and 47.6 × 10^6^ PE reads per sample, while the 3 negative controls yielded 10.3 × 10^6^, 97 × 10^3^ and 82 × 10^3^ PE reads per sample. Positive and negative controls were classified with Kraken 2 using the same approach as with the rumen samples. The positive controls contained 32.4 × 10^6^ and 29 × 10^6^ PE reads classified per sample (62.8% and 60.9% of the raw reads), and the negative controls received 244 × 10^3^, 6 × 10^3^ and 4 × 10^3^ PE reads classified per sample (2.4%, 7.3% and 4.1% of the raw reads). At the genus level, the positive controls contained *Listeria* spp. as the predominant taxa, which was expected according to the mock community composition (ZymoBIOMICS Microbial Community Standard II Log Distribution – Catalog N° 6310). *Bacillus* spp., *Saccharomyces* spp. and *Enterococcus* spp. were above their expected abundances, while *Pseudomonas* spp., *E. coli* and *Salmonella* spp. Were below their expected abundances. All 10 members of the mock community were identified within the positive control samples, including the lowest-abundance taxon *Staphylococcus* spp. The negative controls contained mostly *Butyrivibrio* spp., *Prevotella* spp., *Cutibacterium* spp. and human DNA, which are expected contaminants from rumen samples and human manipulation (Sup. Figure 2).

## Discussion

This study assessed the long-term effect of castration timing and short-term effect of weaning strategy on the rumen microbiome of beef calves using a randomized controlled trial and longitudinal sampling. The dataset alone represents a substantial contribution to the limited body of literature pertaining to the rumen microbiome of beef calves specifically [17, 42]. Using this dataset, we showed that the rumen microbiome shifted as calves approached weaning age, with a very noticeable and rapid change occurring within the first 48 h after weaning. We did not find a significant long-term effect of castration timing on the temporal dynamics of the rumen microbiota (taxonomic profile), although our sampling intervals may not have been frequent enough or close enough to the early castration events to capture short-term differences that may have occurred. Recent studies have found an association between the intestinal microbiome and increased adiposity [43] and growth inhibition [44], both of which can be impacted by the hormonal changes that occur with castration. Given this prior evidence and the limitations of our sampling design, we cannot definitively rule out an association between castration timing and rumen microbiome development, and thus more studies are warranted.

Our study showed that the changes in the rumen microbiome 48 h post-weaning were significantly different in the calves weaned by fence-line compared to those weaned by truck. This effect was also observed when analyzing only the methane-associated genes and microbes of the rumen metagenome (functional profile); specifically, *Methanobrevibacter* and some genes in the hydrogenotrophic methanogenic pathway were in higher relative abundance in calves weaned by fence-line, while some genes in the methylotrophic methanogenesis pathway were in higher relative abundance in truck-weaned calves. In contrast, the rumen resistome not detectably altered by weaning, instead demonstrating a consistent dominance of tetracycline resistance genes across time and treatment groups.

### Rumen microbiota dynamics around weaning could be driven by dietary and stress factors

The overall dominance of the phyla *Bacillota* (synonym Firmicutes) and *Bacteroidota* in the rumen microbiome of beef calves of weaning age was previously reported [28, 45]. Both phyla seem to be dominant throughout rumen development in beef calves [17]. The phyla *Actinomycetota* and *Fibrobacteres* had different relative abundances in previously reported rumen samples collected from beef calves at about the same weaning age; and while the phyla *Verrucomicrobia* and *Tenericutes* represented less than 1% of total abundance in our study, they were present in at least 2% relative abundance in other studies [28, 42, 45]. This heterogeneity in findings may be explained by several factors such as different diets, genetics, and environments. In our study, the phylum *Bacillota* was highly abundant pre-weaning but decreased in relative abundance post-weaning. Although it is not possible to interpret the functionality of any phylum by only discussing a single genus, within the phylum *Bacillota* we can highlight the role of the genus *Butyrivibrio* spp., a hemicellulose degrader and main producer of butyrate, which has been reported as highly abundant in the rumen of calves from birth to 96 days of age [17]. During this same three-week period just prior to weaning, the low abundance (< 1%) phylum *Elusimicrobia* decreased remarkably across all calves. This phylum is an understudied anaerobic bacteria reported to be increased in feedlot finisher cattle [46] and in high-forage-fed dairy cattle during the dry period [47]. As previously described, time-dependent changes of the rumen microbiome have been shown to be influenced by age and diet in cattle [15, 25, 45, 48] and other ruminants [49, 50].

Weaning strategy was associated with immediate differences in the rumen microbiome of the beef calves in this study. Weaning is an important physiological and life cycle event across all mammalian species. It can be especially important in livestock species because it involves not only dietary changes, but often concomitant separation from the dam, social regrouping, transport, and a new environment. The most common strategy for weaning beef calves is abrupt weaning, which involves the immediate separation of the calf from the fam with the relocation of the calf to a new environment, often involving significant transportation. This is recognized as a major stressor for calves and lower stress methods are being explored [51]. This study looked at fence-line weaning as an example of a gradual weaning strategy. Fence-line weaning utilizes an approach of removal of the dam from direct contact with the calf by separation with a sturdy fence and leaving the calf in the environment, most commonly pasture that was previously occupied by both dam and calf. This results in limiting stress by a limited change to diet and environment, other than the removal of direct contact with the dam and the ability to nurse. While it increases weight gain in the calf, it may represent increased feed cost for the operation [51], and is not always possible without appropriate pasture and fencing. The effects of weaning on animal productivity and welfare have been studied extensively [22, 51, 52], but their mechanisms and the impact of different weaning strategies on important phenotypes and production outcomes are still being investigated. The impact of weaning on the gastrointestinal microbiome is gaining attention since it has been described in swine [53], horses [54], sheep [55], goats [50], dairy cattle [33], and recently in beef cattle [17, 42].

Consistent with previous studies in cattle [33] and other livestock species [49, 53], we observed a significant decrease in rumen microbial diversity shortly after weaning, which was especially marked in truck-weaned calves. Stressors such as heat [56] and long-distance ground transportation [57] have also been associated with decreased rumen microbiome diversity in cattle, suggesting that physiological stressors at the host level may also manifest as decreased diversity within host-associated microbiomes. Interestingly, the rumen of truck-weaned calves had the lowest microbial diversity across all collection days and groups, which may be explained by the additional stressors these calves experienced due to ground transportation, physical separation from their dams, and change of environment as well as diet. The mechanisms involved in the response of gastrointestinal microbiomes to physiological stressors are still unclear but may include: oxidative stress, erratic activation of immune response against bacteria and the secretion of bacterial toxins [58–60]. To note, our study did not measure stress levels using biological markers and thus we did not present our results in terms of “high” or “low” stress weaning methods. However, the body of evidence shows that sequential weaning (i.e., via fence line) is associated with decreased levels of stress-associated biomarkers compared to abrupt weaning by long-distance transportation [22, 51].

We observed a significant increase in the relative abundance of the 2 most abundant genera, *Fibrobacter* spp. and *Prevotella* spp., in the 48 h after weaning. *Fibrobacter* spp. are cellulose degraders previously identified as part of the core heritable rumen microbiome [14] that colonizes the rumen of beef calves after 7 days of age [17]. The transition of calves to a forage- or grass-exclusive diet may provide a fiber-rich substrate that supported the relative increase of this genus with the rumen microbial community. The increase of *Prevotella* after weaning and during dietary transitions has been described in cattle [15, 33, 48], pigs [53] and horses [54]. This genus is composed of several species with a variety of biological functions, including use of readily available carbohydrates, degradation of hemicellulose, and protein and peptide breakdown [61, 62]. The wide metabolic plasticity of the genus *Prevotella* offers evidence of the complex functional profile of the rumen microbiome. For instance, some *Prevotella* species have been associated simultaneously with both low and high methane emission cattle [63], while other species such as *Prevotella ruminicola* have been identified as the bacterial host of antimicrobial resistance genes in the cattle rumen [64]. Interestingly, increases in the abundance of this genus in the rumen have also been associated with other non-dietary stressors in cattle [35, 65], suggesting a role of microbes in the physiological response of cattle to diverse stressors. It is challenging to identify a single or definitive mechanistic link between rumen microbiome variation and host-level stressors such as a change of diet and weaning. In humans, the gut microbiome is considered part of the gut-brain axis, which helps regulate stress through processes that include vagus nerve modulation, gut hormone signaling, the immune system and microbial metabolites [66]. The role of rumen microbes in the stress response of ruminants is still unclear and requires further study.

Weaning as a process represents numerous changes (i.e., diet, age and change of environment), all of which can impact both the bovid host and its associated microbes. Our study was not designed to isolate the effect of each factor, but instead to describe the total effect of typical weaning processes as a multi-factor event that occurs during the cattle life cycle. Dietary shifts and aging are co-occurring factors that affect the rumen microbiome and are nearly impossible to disentangle. Nonetheless, we tried to limit the potentially confounding effect of other covariates such as pre-weaning diet and genetic background, while also appropriately mimicking two common weaning practices in the US [52], as they happen in the field. Our study did not include long-term sampling to understand the long-term effects of weaning on the rumen microbiome. If they exist, such long-lasting shifts in the rumen microbiome may be highly relevant to important animal health and production outcomes, and thus should be addressed by future studies.

### Ruminal methane-associated genes may be more readily influenced by external factors than antimicrobial resistance genes

Methane is produced in the rumen as one of the end products of the fermentation process [16]. Many microbes from the archaeal community of the rumen, particularly the phylum *Euryarchaeota*, are associated with methane production (e.g., *Methanobrevibacter Gottschalkii and Methanobrevibacter Ruminantium)*. These microbial taxa are found in the rumen microbiome from an early age and may be part of the initial microbial colonization of the rumen [17, 67]. In a variety of studies, a set of non-archaeal microbes have also been associated with high methane emission in cattle: *Christensenellaceae, Mogibacteriaceae, Ruminococcaceae, Lachnospiraceae and Rikenellaceae* [9, 68–70]. On the other hand, several others are highly abundant in low methane emitters: *Methanosphaera, Vellionellales* and *Desulfovibrionales* [9, 71]. The literature in this area is still somewhat ambiguous, but previous findings combined with environmental evidence have allowed the development of a preliminary database of methane-associated bacteria, archaea and genes [39]. Using this tool, we observed a significant association between weaning strategy and the composition of methane-associated genes in the rumen of beef calves. Rumen methanogenesis uses 3 pathways: hydrogenotrophic, methylotrophic and acetoclastic [3, 16]. Our results suggest that at 48 h post-weaning, the rumen microbiome of calves weaned by fence-line have more capacity to generate methane via the hydrogenotrophic and central pathways but lower potential capacity to generate it via the acetoclastic and methylotrophic pathways. Considering that hydrogenotrophic methanogens are the most abundant in cattle rumen [16], our results suggests that a higher methanogenesis capacity via the main pathway may functionally contrast with lower methanogenesis capacity via alternative pathways in fence-line-weaned calves (Fig. 5). Although methane oxidation (i.e., methane use) pathways are not extensively studied in the rumen, there is evidence of methane oxidation capacity [72] and the presence of methanotrophs in the rumen of cattle [73] and other ruminants [74]. Due to the anaerobic environment of the rumen, it is thought that only anaerobic methanotrophs inhabit the rumen [75]. However, an oxygen flow in the rumen has been described [76], and thus aerobic oxidation of methane cannot be definitely ruled out, especially in the highly oxygenated rumen epithelium [72, 74]. We provided DNA-level evidence that aerobic and anaerobic methane oxidation genes are present in the rumen metagenome and that they are impacted by weaning strategy, with calves weaned by fence-line containing less potential capacity to oxidate methane via aerobic and anaerobic pathways. In addition, we found the genus *Methanobrevibacter* in higher relative abundance in fence-line-weaned calves compared to truck-weaned calves. Altogether, our results suggest that the rumen microbiome of calves weaned by fence-line may have an increased capacity to generate methane due to higher abundance of methanogens, higher hydrogenotrophic methanogenesis capacity and lower methane oxidation capacity, when compared to calves weaned by truck, at least in the very immediate post-weaning period.

However, we caution against over-interpretation of these results, particularly because our analysis is conducted at the DNA level, which does not necessarily correlate with transcription and production of metabolites such as methane. Proper correlation with metatranscriptomics, proteomics, metabolomics and/or phenotypic testing remains to be elucidated in future studies. Additionally, more extensive phenotypic measurements would provide more actionable results, including respiratory chambers or antimicrobial susceptibility testing to measure methane and phenotypic antimicrobial resistance, respectively. Since these metrics are beyond the scope of this study, the reader should not extrapolate our DNA-level results to phenotypic expression. Moreover, metagenomic studies require replication, particularly given the relatively sparse information and sequence databases available for methane-production pathways and microbes. Given that methane production is typically the result of complex gene-gene interactions, the results generated from this gene-by-gene approach need further validation. A rumen-specific methane metabolism database with comprehensive annotations is a critical gap for future metagenomic studies of the rumen microbiome and methane production.

Recent evidence suggests that the rumen can be a potential source of antimicrobial resistance genes, with a highly diverse and concentrated microbial community that can favor horizontal gene transfer [37, 77, 78]. Our study found a very consistent and dominant distribution of tetracycline (*tet40, tet44, tetO, tetQ, tetW*) and nitroimidazole (*nimJ*) resistance genes within the rumen resistome. Our findings regarding the dominance of tetracycline resistance genes are consistent with previous studies of the rumen resistome of dairy cattle, both at the DNA [79, 80] and RNA levels [78]. Furthermore, this pattern of a tetracycline-dominated resistome has been described in numerous beef and dairy resistome studies investigating different sample types (e.g., feces, soil and water) and even different countries [81–83]. Contrasting to our results, chloramphenicol, microcin, aminoglycoside and streptomycin resistance genes have been reported to be more prevalent in the rumen of adult beef cattle not exposed to antibiotics [37]. While concentrate-based diet [37] and even milking traits [80] have been associated with differences in the rumen resistome, we did not observe an association with age, castration timing or weaning strategy in beef calves. Interestingly, the predominant tetracycline resistance pattern in the rumen is reflected in feces, as described not only in dairy calves [84] but also in unexposed wild ungulate species (elk and bison). The presence of tetracycline resistance genes in wild ruminants suggests that this phenomenon may have broader origin in wild animals [85].

The most abundant gene across all samples in this study was *tetW*. Recently, a high abundance of *tetW* transcription within the rumen of beef cattle was reported using a metatranscriptomics approach; and the carbohydrate degraders *Ruminococcus* spp., *Prevotella ruminicola*, *Muribaculaceae* spp. and *Collinsella aerofaciens* were listed as common bacterial hosts of expressed ARGs [64]. Additionally, the highly abundant *tetW* gene has been found located in a novel integrative and conjugative element in the ruminal community [78], supporting the hypothesis that horizontal gene transfer of ARGs within the rich and complex microbial community of the rumen supports the abundance and persistence of *tetW*. Further research is needed to both replicate this finding and to understand its importance, considering the rumen microbiome of cattle not only as a potential source of ARGs [37], but also as a potential ecosystem favorable to increased horizontal gene transfer [16].

### Unclassifiable sequence reads dominated the rumen metagenomic data

The use of metagenomics in microbiome research has drawn attention to the high amount of still-unidentified genomic material that comprises many microbial communities [86]. The proportion of unclassified sequences, referred to as microbial dark matter [86] or dark microbiome [87], varies depending on the niche. Between 25 and 81% [86, 88] of metagenomic sequence data from diverse environmental niches has been cataloged as unknown; in potentially less diverse niches, the proportion of sequences that remain unclassified can be lower. For instance, around 50% of non-host sequences have been reported as unclassified in the microbiome of *Arabidopsis thaliana* leaves [89], while 2–4% of sequences in industrial food ingredients are reported as unknown [90]. Although well documented, this limitation is scarcely reported and addressed in rumen microbiome research. From 22 rumen microbiome studies that cited Kraken [91] or Kraken 2 [38] as the taxonomic classifier in PubMed (accessed on April 2023), only 5 addressed the classification rate issue [41, 92–95]. Three of the 5 studies showed that expanding the taxonomic reference database (either through inclusion of the Hungate project genomes [96] and/or metagenome-assembled genomes [MAGs]) increased the classification rate of rumen metagenomic data up to 50% [94], 62.6% [92] and 70% [95]. Despite the substantial improvement in classification rate obtained with the inclusion of MAGs, a recent study reported that incomplete or informal taxonomic lineages for the MAGs (i.e., lack of appropriate labels at every taxonomic rank) can greatly limit their classification at lower taxonomic levels (i.e., genus or species) [41]. Interestingly, another one of these 5 studies identified on average 12% of the previously unclassified metagenomic reads as ciliates by adding 52 high-quality rumen ciliate genomes to their reference database [93]. Altogether, these efforts highlight the importance of reference database customization with rumen-specific organisms (bacterial and non-bacterial) to increase the classification rate, especially given the relatively low classification for metagenomic rumen data. For this reason, we increased our classification rate while also ensuring accurate genus-level classification by customizing our reference database with the genomes of the Hungate project and the most comprehensive collection of archaea, bacteria, virus, plasmids, human, UniVec_core, protozoa and fungi from RefSeq NCBI. However, our classification rates still remained inconsistent to those reported by previous rumen microbiome studies [92, 94, 95]. The difference in host demographics could explain this discrepancy, as previous metagenomic rumen datasets were generated from samples obtained from dairy and/or mature cattle; while our data originated from immature beef cattle. However, systematic and formal comparisons are needed to understand the reasons for this discrepancy in classification rates despite very similar custom databases and bioinformatic workflows.

Many rumen researchers have responded to the recognized need to improve classification rates for metagenomic rumen datasets. Current efforts include the Hungate project [96], exploring the yet-unknown microorganisms of the rumen through culturomics [97], and even more, investigating the neglected viral [98] and plasmid [99] communities of the rumen. Until classification rates are significantly improved, there is risk of serious bias in rumen metagenomic analyses, which limits our confidence in interpreting not only the taxonomic profile of rumen microbial communities, but also their functional potential [100]. Thus, an extensive exploration of the rumen metagenome is required to corroborate that the shifts in alpha and beta diversity we observed in the classified portion of the rumen metagenomic DNA accurately reflects the shifts occurring in the unclassified portion. Likewise, further research is needed to understand the association between taxonomic classification rate and accuracy of observing changes in microbiome diversity and taxonomic profiles in the rumen.

## Conclusion

The rumen microbiome of beef calves is a complex and dynamic community that shifts around weaning. We observed that differences in weaning strategy were associated with significant differences in the rumen microbiota (taxonomic profile) and rumen metagenome (functional profile) 48 h after weaning. Unlike methane-associated genes, ARGs were not significantly impacted by weaning nor weaning strategy. Additionally, castration timing did not significantly alter the rumen microbiota in the long term, although our sampling timeline precluded observation of potential short-term differential impacts. More studies are warranted to describe the short-term effects of castration timing and long-term effects of weaning strategy on the rumen microbiome of beef calves, and to understand whether any differences substantially impact health and production outcomes. Further work is also needed to build up existing reference databases to improve taxonomic classification rates specifically for beef calf rumen microbiomes.

## Methods

### Study design and sample collection

#### Animal handling and interventions

The aim of this study was to evaluate the impact of castration timing and weaning strategies on the rumen microbiota (taxonomic profile) and the metagenome (putative functional profile) associated with methane emission and antimicrobial resistance (AMR) in the rumen.

We conducted a randomized controlled trial in a single cow-calf herd at the North Central Research and Outreach Center (NCROC) at the University of Minnesota (Grand Rapids, MN) from March to October 2021. The source herd comprised approximately 120 certified Angus cows raised on 801,278 m^2^ of mixed pasture. All bull calves born in the 2021 season were eligible for study enrollment unless they were born under dystocia conditions, with a visible abnormality or disease. We consecutively enrolled 32 male calves at birth over a period of 26 days during the 2021 calving season (March – April). Our study assessed two management interventions in a 4 × 2 factorial design: 4 different castration timings and 2 weaning strategies. A random number generator was used to determine the order of treatment allocation, and animals were allocated sequentially at birth to both interventions with a balanced and crossed design, with 8 animals per each castration timing group and 16 animals per weaning strategy group. Sample collection and animal handling were done following ethical guidelines approved by the Institutional Animal Care and Use Committee (IACUC) of the University of Minnesota, protocol ID: 2102-38861 A.

The study calves had similar genetic backgrounds, were fed the same diet, were turned out in a single group and, except for castration timing, managed under the same standard procedures until weaning. Calves were exclusively nursing from birth to turnout on grass and were in a full-time pasture regime from turnout to weaning. They were rotationally grazed on 150 acres split up into 6-acre paddocks. Each pasture was supplied with free-choice mineral feeders containing a mixture of salt and wind and rain fly control mineral that was available to all animals. As part of the health management plan, several dewormers and vaccinations were administered to the study calves on the same days as the castrations, as follows: at turnout Ultrachoice® 8 (Zoetis, USA), Inforce® 3 (Zoetis, USA), Nuplura® PH (Elanco, USA) vaccines and Cydectin® dewormer (Elanco, USA); at pre-weaning Titanium® 5 (Elanco, USA), Nuplura® PH (Elanco, USA) and Ultrachoice® 8 (Zoetis, USA) vaccines; at weaning Valbazen® dewormer (Zoetis, USA), Titanium® 5 (Elanco, USA), Nuplura® PH (Elanco, USA) and Ultrachoice® 8 (Zoetis, USA) vaccines. Since study calves were part of a commercial herd, the weaning day was selected following common criteria for cow-calf operations in Minnesota; such criteria included the availability of pasture and other feeds, the body condition of cows, and market availability for the calves. Study calves were monitored closely by NCROC staff, who reported any variation in diet, behavior, or health issues.

The 4 castration timing groups assessed were: castration within 48 h of birth (March 14th – April 9th), castration at turnout (average of 64 days after birth, i.e., 4 months before weaning, on May 25th), castration at pre-weaning, (average of 180 days after birth, i.e., one month before weaning, on September 21st) and castration at weaning (average of 208 days after birth, on October 18th) (Fig. 1). Birth and turnout castrations were done using the Ideal® Calf and Lamb Bander (Neogen, USA). Briefly, the testicles and scrotum were pulled down, the band was opened and placed up over the scrotum, and after checking that the testicles were still in the scrotum, the band was released just above the top of the testicles. A final check was done to ensure both testicles were still in the tip of the scrotum and that the ring was placed properly. Pre-weaning and weaning castrations were performed in the chute located at the handling facility before collecting rumen samples. The procedure was similar to the birth and turnout castrations, but an XL Castrating Bander (Wadsworth Manufacturing, USA) was used. All animals, regardless of their castration group, were checked for testicles at pre-weaning and weaning collection days.

Since castration was performed at different time windows, the castration groups varied depending on the collection day: 3 castration strategies were contrasted at pre-weaning (birth, turnout and not castrated); 4 at weaning (birth, turnout, pre-weaning and not castrated); and 4 at post-weaning (birth, turnout, pre-weaning and weaning) (Fig. 1).

The 2 weaning strategies assessed were weaning by fence-line (N = 16) and truck (N = 16). On the day of weaning, calves were processed and sampled in a chute, and then the calves assigned to the “fence-line weaning” strategy exited the chute and were placed in the same pasture as before weaning. This pasture adjoined a pasture housing their dams, but with separation via an electric fence; both pastures contained a mix of Kentucky bluegrass (*Poa pratensis*), tall fescues (*Festuca arundinacea*), red clover (*Trifolium pratense*), timothy grass (*Phleum pratense*), orchard grass (*Dactylis glomerata*), perennial ryegrass (*Lolium perenne*), smooth brome (*Bromus inermis*), with no extra dietary supplementation. Calves assigned to the “truck weaning” strategy exited the chute and were assembled in a preloading pen and then loaded onto a truck and transported for 2 h. After transport, they were unloaded into a feedlot-sized pen at the south station of NCROC, where they were kept in a roofed pen with a thick layer of straw bedding and a J-bunk concrete feeder. They were given a moderate quality 25/75 alfalfa to grass mix, supplemented by Wind and Rain® mineral and American Stockman® salt. This formulation was intended to better reflect the pasture-based diet of the fence-line weaning group, i.e., a grass-based diet without concentrate-based supplementation.

#### Sample collection and weighing

Ruminal samples were collected on 3 collection days: at pre-weaning (one month before weaning, on September 21st); at weaning (right before they were weaned, on October 18th); and at post-weaning (2 days after weaning, on October 20th) (Fig. 1). Weaning is defined as the time at which calves are physically separated from their dams, and unable to obtain milk.

For each collection day, all study calves were run through the chute and rumen fluid was collected by esophageal tubing using a Frick’s speculum adapted to a collection flask and vacuum pump. Between each animal, the tubing and collection flask were emptied, disinfected with sodium hypochlorite at approximately 10% concentration, and rinsed thoroughly with tap water. The tube was inserted into the oral cavity and advanced down the esophagus until the fiber mat was reached, at which point the tube was retracted 5–8 cm in order to obtain fluid. Rumen fluid was collected in 50 ml sterile tubes, immediately transported to the laboratory at approximately 4 °C and stored at -80 °C within 4 h of collection. Rumen fluid was processed without filtering at any step.

Each calf was weighed using a floor scale (Tru-test, USA) adapted to the cattle chute, at birth, turnout and on the 3 days of sample collection. The average daily gain (ADG) was obtained individually for each animal, subtracting the birth weight from the post-weaning weight and dividing the result by the age in days.

Once data were collected, we used Kruskal-Wallis or ANOVA test to assess statistical significance of weight at birth, average daily gain and age between castration and weaning groups to determine whether to include them as potential covariates in further regression models.

### DNA extraction and sequencing

DNA was extracted from each sample in randomized batches of 12 samples to avoid collinearity between batch effect and treatment group. Samples were processed under aseptic conditions to avoid cross-contamination. DNA extraction blanks consisting of CD1 solution (lysis buffer provided in the DNA extraction kit) were used as negative controls. To begin DNA extraction, rumen fluid was thawed and homogenized by vortex for 3 min. An aliquot of 1 ml was centrifuged at 16 000 rcf for 10 min in an Eppendorf 5415R centrifuge at room temperature. Supernatant was discarded, and the remaining pellet was used as initial sample for column-based DNA extraction using the Dneasy® PowerSoil® Pro Kit (QIAGEN, USA) following the manufacturer’s protocol without modifications. Briefly, the pellet was resuspended in 800 µl of CD1 solution (lysis buffer), suspension was transferred to PowerBead Pro® Tubes (zirconium beads), then bead beating was performed in 3 cycles of 20 s at 2,200 rpm with 30 s intervals and centrifuged at 16 000 rpm for 2 min. Finally, 600 µl supernatant were transferred to QIAcube Connect® equipment (QIAGEN, USA) for a fully automated DNA extraction.

DNA extractions were submitted to the University of Minnesota Genomics Center (UMGC). Along with sample DNA and negative controls, mock community DNA (ZymoBIOMICS Microbial Community Standard II Log Distribution – Catalog N° 6310) already extracted following the process described above, was included as a positive control for the library preparation and sequencing process. DNA quantity and quality was assessed using the PicoGreen assay (Thermo Fisher, USA) and 260/230 ratio in Nanodrop1000 (Thermo Fisher, USA), respectively. Barcoded libraries were generated using Illumina Nextera XT DNA library preparation kit (Illumina, USA) following manufacturer’s protocol. Shotgun metagenomic paired-end sequencing (2 × 150 bp) was performed in a single pool across 2 lanes of S4 flow cells (2,250 million reads/lane expected) of a NovaSeq 600 platform (Illumina, USA) using kit v1.5 (300 cycles).

### Microbiota analysis

#### Bioinformatics

Demultiplexed paired-end sequencing reads were analyzed using the AMR + + v2.0 pipeline [40]. This suite includes quality-based trimming and filtering of sequencing reads using Trimmommatic [101], alignment of surviving high-quality reads to the host genome using Burrows-Wheeler-Aligner (BWA) [102], removal of host-aligned reads by SAMtools [103], file format conversion using BEDTools [104], antimicrobial resistance genes aligning using MEGARes 2.0 database [40] and microbiome taxonomic profiling using Kraken 2 [38].

Specifically for taxonomic profiling (microbiota analysis), we initially followed an standard protocol: (1) host-decontamination aligning high-quality reads to *Bos taurus* reference genome (Genome Bos_taurus_UMD_3.1, accession number: GCA_002263795.3), (2) taxonomic classification with Kraken 2 [38] using a confidence score of 0 and the standard genome database (accessed in July 2020), which contained archaea, bacteria, virus, plasmids, UniVec_Core and the human genome. As this protocol was not able to classify a high proportion of sequencing reads, we attempted a classification rate increase by a customized rumen-specific protocol detailed as follows: (1) For the host-reads decontamination step we aligned the high-quality reads to *Bos taurus* reference genome (Genome Bos_taurus_UMD_3.1, accession number: GCA_002263795.3) and dietary plants genomes available in GenBank, which included: *Trifolium pratense* (full genome, accession: ARS_RC_1.1), *Dactylis glomerata* (full genome, accession: GCA_007115705.1), *Lolium perenne* (full genome, accession: MPB_Lper_Kyuss_1697), *Poa pratensis* (chloroplast genome, accession: NC_057962.1), *Festuca arundinacea* (chloroplast genome, accession: NC_011713.2), *Phleum pratense* (chloroplast genome, accession: NC_067044.1), *Bromus inermis* (chloroplast genome, accession: NC_067047.1), and *Festuca pratensis* (plastid genome, accession: NC_019650.1); (2) taxonomic profiling using Kraken 2, with a confidence parameter at 0.1 to decrease the likelihood of spurious (i.e., false positive) classifications [38]. Instead of using the default Kraken 2 database, we built a custom database that included reference genomes from RefSeq’s NCBI for archaea, bacteria, virus, plasmids, human, UniVec_core, protozoa and fungi (accessed in January 2023). In addition, we added genomes obtained from 410 rumen-specific bacteria isolated for the Hungate project [96] (source: https://genome.jgi.doe.gov/portal/pages/dynamicOrganismDownload.jsf?organism=HungateCollection). It was previously shown that including these genomes significantly increase the taxonomic classification of rumen microbiome samples [41].

Results from microbiota analysis by Kraken 2 [38] were printed for all samples in a count matrix using python scripts included in the AMR + + v2.0 suite [40]. The count matrix contained taxonomic information for each Operational Taxonomic Unit (OTU) and counts of each OTU (as rows) for each sample (as columns).

Once sequencing and taxonomic classification data was obtained, we used Kruskal-Wallis or ANOVA tests to assess statistical significance of number of raw sequencing reads, number of non-host reads and number of classified reads between castration and weaning groups to determine whether to include them as potential covariates in further regression models.

#### Data analysis and statistics

The microbiota analysis was conducted in terms of the collection day, castration timing groups and weaning strategy groups. The OTU count matrix obtained by the bioinformatic analyses, and the study metadata, were used for microbiota data analysis and statistics in R (version 4.1.0, https://www.r-project.org/). Using *phyloseq* v1.4 package [105], we combined metadata, the count matrix and taxonomic information to build a *phyloseq* object. We normalized our count matrix using the Cumulative Sum Scaling (CSS) method in the *metagenomeseq* v1.4 package [106]. Our downstream analysis was focused on the bacterial and archaeal domains due to their major role in ruminant metabolism (biological importance) and the high proportion of classified reads that they represented among total reads (technical importance). We filtered our dataset to keep only bacteria and archaea domains and agglomerated them to phylum and genus taxonomic ranks using *phyloseq* v1.4. The relative abundance of OTUs, considered as the proportion of counts for a given OTU out of the total OTU counts in the sample (usually in the 0-100% scale), were plotted using *ggplot2* v3.4.1 [107] and *phyloseq* v1.4.

For the alpha diversity analysis, considered as the diversity within the sample, we calculated Richness and Shannon’s index for each rumen sample at the genus level using *phyloseq* v1.4 the non-normalized dataset. Richness is an indicator of number of unique OTUs in the sample, while Shannon’s index is an indicator of richness and evenness of OTUs in the sample. The statistical analysis of each index was done separately using *lme4* v1.1-35.1 [108] and *lmerTest* v3.1-3 [109] packages. The index was included as dependent (outcome) variable in a linear mixed-effect model, in which the independent (predictor) variables were animal ID as random effect (to account for repeated measures over time on each calf), and the interaction term of weaning strategy and collection day, castration group, age and weight as fixed effects. Type-III ANOVA was used to assess statistical significance for each independent variable in the model using *car* v3.1-2 [110]. Unless otherwise stated, statistical significance was considered at a *P* < 0.05. When statistically significant, a post-hoc comparison was done between groups with least square means using the package *emmeans* v1.8.9 [111]. Alpha diversity indices were plotted as boxplots stratified by collection day and weaning strategy using *ggplot2* v3.4.1.

For the beta diversity analysis, considered as the diversity between samples, we used the CSS-normalized count matrix at the genus level. The ordination analysis and beta diversity plots were done with *phyloseq* v1.4 using the non-metric multidimensional scaling (NMDS) method and Bray-Curtis distances. For the statistical analysis, we calculated the Bray-Curtis dissimilarity matrix for the CSS-normalized count matrix at the genus level using *vegan* v.2.6-4 [112]. We performed a Permutational Multivariate Analysis of Variance (PERMANOVA) using *vegan* v.2.6-4. The distance matrix was included as dependent (outcome) variable and weaning strategy, collection day, and castration group as independent (predictor) variables in a marginal model (option “by = margin” in *adonis2* function). The R^2^ value was used as the proportion of variation explained by a given independent variable and the associated *P* value was used to determine statistical significance. Unless otherwise stated, statistical significance was considered at a *P* < 0.05.

We performed a differential abundance testing to identify specific OTUs that differ in abundance between 2 group of samples using *metagenomeseq* v1.4 [106]. We addressed the sparsity of the data by using the CSS-normalized datasets (at the phylum and genus level). We accounted for typical excess of zeros in microbiome data by using a multivariate zero-inflated gaussian mixture model, which estimates the probability that a zero for a particular feature is generated from the detection distribution (technical zero) or from the count distribution (real zero) [106]. Our model used animal ID as random effect and weaning strategy, collection day and castration group as fixed effects. In addition, we addressed poor control of false discovery rates, usually claimed as an issue of non-compositional methods for differential abundance testing, by using the Benjamin-Hochberg (BH) *P* value correction for multiple pairwise comparisons of the log2-fold change between groups and by reporting the OTUs with average log2 abundance across sample > 5 and log2-fold change > 0.5 or < -0.5.

### Metagenomic analysis

#### Methane associated genes and microbes

The bioinformatic analysis for methane-associated genes was done using the functional profiling scripts and database of McyCDB [39, 113], with default settings and non-host reads as input. Briefly, the forward and reverse non-host reads were merged using PEAR [114] and then a translated search in the McycDB database was done using DIAMOND [115] with e-value = 1e-4. For methane-associated genes analysis, the number of sequences in every sample was normalized by random subsampling to 6,188,129 reads per sample (the minimum number of sequences per sample within our dataset) for downstream analysis. The functional profiling results were reported at the gene level. We defined methane-associated genes as genes involved in methane cycling pathways either in the central methanogenic pathway, hydrogenotrophic methanogenesis, acetoclastic methanogenesis, methylotrophic methanogenesis, anaerobic or aerobic oxidation of methane [39]. As McycDB does not offer gene ontology, we manually annotated our results with pathway information from the article [39] and stratified our results by methane cycling pathways.

The bioinformatic analysis for methane-associated microbes was done using the taxonomic profiling scripts of McyCDB [39]. In addition to the previously described steps, the merged reads matching a methane-associated gene were extracted using SeqKit [116] and taxonomically classified using Kraken 2 [38]. The taxonomic profiling results were reported at the genus level. We defined methane-associated genera as organisms that are involved in the methane cycling pathways mentioned above [39]. Unfortunately, a differentiation of microbes involved on each pathway was not possible as McycDB does not provide that stratified information.

The methane-associated gene and microbe data analysis was performed in terms of the collection day and weaning strategy groups. For the data analysis and statistics of the methane-associated genes in R v4.1.0, we combined the metadata, the count matrix and the gene name information to build a *phyloseq* object using *phyloseq* v1.4. We normalized our count matrix using the Cumulative Sum Scaling (CSS) method in *metagenomeseq* v1.4 package. The analysis of methane-associated microbes followed similar steps but used the genus name information instead to build the *phyloseq* object.

For the alpha diversity analysis of methane-associated genes, we calculated the Shannon’s index of each sample at the gene level using *phyloseq* v1.4. For statistical analysis, we used *lme4* v1.1-35.1 and *lmerTest* v3.1-3 packages and the same model evaluated in the microbiota data analysis but considering the Shannon’s index of methane-associated genes as dependent variable. Type-III ANOVA test was conducted, and least square means were obtained using *car* v3.1-2 and *emmeans* v1.8.9, respectively. Alpha diversity indices were plotted as boxplots stratified by day and weaning strategy using *ggplot2* v3.4.1. For the beta diversity analysis, we followed a similar procedure as stated in microbiota analysis section but using the CSS-normalized count matrix of methane-associated genes at the gene level as input of the ordination analysis with *phyloseq* v1.4 and distance matrix calculation with *vegan* v2.6-4.

Only the relative abundance of methane-associated microbes was plotted at the genus level using *ggplot2* v3.4.1 and *phyloseq* v1.4. The differential abundance testing was conducted for genes and genera with *metagenomeseq* v1.4, following the step detailed in microbiota analysis but using only samples from at weaning and post-weaning collection day. Only features (genes and genera) with average log2 abundance across sample > 5 and log2-fold change > 0.5 or < -0.5 were listed. The results of methane-associated genes were stratified by methane cycling pathway.

#### Antimicrobial resistance renes (resistome)

The bioinformatic analysis for antimicrobial resistance gene (resistome) was done using implemented scripts on AMR + + v2 pipeline. Non-host reads were aligned to the MEGARes 2.0 database [40] using BWA [102]. Antimicrobial resistance genes (ARGs) that obtained at least 80% gene fraction were considered present in the respective sample. All aligning reads were counted and printed for all samples in a count matrix using python scripts included in the AMR + + v2.0 [40]. The count matrix contained ontology information for each ARG at 4 levels (Type, Class, Mechanism and Gene), but all the analysis were conducted only at the gene level. ARGs labeled as ‘*RequiresSNPConfirmation’* were excluded from the count matrix and not considered for downstream analysis.

The ARG analysis was performed in terms of the collection days and weaning strategy groups. For the statistical analysis, a *phyloseq* object was built in *phyloseq* v1.4, as stated in previous sections, using the count matrix, ARGs ontology information and study metadata. The *phyloseq* object was normalized by CSS method using *metagenomeseq* v1.4, then agglomerated to the gene level and their relative abundance were plotted using *ggplot2* v3.4.1 and *phyloseq* v1.4. The differential abundance testing at the gene level was conducted with *metagenomeseq* v1.4, using the CSS-normalized data only from at weaning and post-weaning collection day.

### Electronic supplementary material

Below is the link to the electronic supplementary material.

Supplementary Material 1: Contains supplementary tables S1-S5 and supplementary figures 1-5

## Data Availability

The raw sequence data generated during this study are available in the NCBI repository under BioProject PRJNA983521. The code used for data processing, bioinformatic and statistical analysis is stored in the GitHub repository: https://github.com/TheNoyesLab/Beef_calf_rumen.
