# Supplementary material for: Effect of castration timing and weaning strategy on the taxonomic and functional profile of ruminal bacteria and archaea of beef calves
Source: Anim Microbiome. 2023 Dec 1;5:61. doi: 10.1186/s42523-023-00284-2 (PMC10691087; doi:10.1186/s42523-023-00284-2)
Supplement: Supplementary file 1 — Supplementary Material 1: Contains supplementary tables S1-S5 and supplementary figures 1-5 [file 42523_2023_284_MOESM1_ESM.docx]

**SUPPLEMENTARY MATERIAL**

**Supplementary table S1. Number of unique OTUs identified at different taxonomic levels**

| **Domain/sub-kingdom** | **% of total classified reads*** | **Phylum** | **Class** | **Order** | **Family** | **Genus** | **Species** |
| --- | --- | --- | --- | --- | --- | --- | --- |
| Archaea | 1.365 | 7 | 18 | 31 | 48 | 140 | 351 |
| Bacteria | 98.589 | 40 | 93 | 219 | 519 | 1909 | 8674 |
| Other classification * | 0.046 | - | - | - | - | - | - |

(*) Virus, protozoa, fungi or Human reads.

**Supplementary Table S2. List of significantly differentially abundant genera between fence-line and truck weaned calves at post-weaning day (reference group: fence-line). Only genera with logFC > 0.5 or < -0.5 and average abundance > 5 are listed.**

| **Genus** | **logFC** | **Average abundance** | **adj.P.Val** |
| --- | --- | --- | --- |
| *Rhodococcus* | 2.09 | 6.86 | 1.17E-15 |
| *Agrobacterium* | 3.14 | 7.16 | 4.46E-13 |
| *Anaerovibrio* | 2.77 | 7.90 | 1.52E-09 |
| *Oribacterium* | 2.43 | 12.15 | 7.35E-13 |
| *Plantibacter* | 2.42 | 5.87 | 9.91E-13 |
| *Lachnoclostridium* | 2.14 | 9.30 | 3.09E-10 |
| *Variovorax* | 2.52 | 5.21 | 1.32E-11 |
| *Mogibacterium* | 1.26 | 5.56 | 9.50E-11 |
| *Selenomonas* | 1.99 | 12.67 | 1.69E-09 |
| *Microbacterium* | 1.86 | 7.98 | 3.92E-12 |
| *Eubacterium* | 1.60 | 10.15 | 1.53E-08 |
| *Arthrobacter* | 0.91 | 5.49 | 9.17E-09 |
| *Succiniclasticum* | 1.28 | 13.40 | 1.49E-04 |
| *Stenotrophomonas* | 0.99 | 7.24 | 6.40E-04 |
| *Mycolicibacterium* | 1.06 | 6.50 | 2.73E-07 |
| *Butyrivibrio* | 0.95 | 14.36 | 1.30E-06 |
| *Berryella* | 0.76 | 5.22 | 6.61E-06 |
| *Nocardioides* | 1.03 | 6.62 | 8.16E-06 |
| *Pseudomonas* | 0.72 | 9.66 | 1.76E-02 |
| *Rhizobium* | 0.80 | 5.55 | 2.12E-05 |
| *Sodaliphilus* | 0.75 | 6.02 | 3.47E-05 |
| *Methanobrevibacter* | 0.71 | 11.41 | 4.71E-04 |
| *Bradyrhizobium* | 0.96 | 6.23 | 8.03E-05 |
| *Vescimonas* | 0.55 | 7.31 | 5.93E-04 |
| *Lachnobacterium* | 0.73 | 6.20 | 5.24E-03 |
| *Kandleria* | 1.65 | 6.96 | 6.15E-03 |
| *Methylorubrum* | 0.56 | 5.27 | 1.06E-02 |
| *Methylobacterium* | -0.69 | 8.50 | 3.88E-03 |
| *Bacteroides* | -1.01 | 11.27 | 6.18E-05 |
| *Lachnospira* | -1.06 | 7.76 | 7.29E-03 |
| *Petrimonas* | -0.56 | 5.00 | 8.60E-03 |
| *Micromonospora* | -0.53 | 6.09 | 1.05E-02 |
| *Fibrobacter* | -1.12 | 14.26 | 1.23E-02 |
| *Sarcina* | -1.21 | 12.07 | 9.07E-06 |
| *Ruminococcus* | -1.38 | 13.07 | 3.80E-08 |
| *Streptococcus* | -2.31 | 9.85 | 6.41E-13 |

**Supplementary Table S3. List of significantly differentially abundant methane-associated genera and genes between fence-line and truck weaned calves at post-weaning day (reference group: fence-line). Only features with logFC > 0.5 or < -0.5 and average abundance > 5 are listed.**

| **Feature** | | **Gene pathway** | **logFC** | **Average abundance** | **adj.P.Val** |
| --- | --- | --- | --- | --- | --- |
| Genera |  |  |  |  |  |
|  | *Methanobrevibacter* | - | 0.78 | 10.68 | 1.2E-05 |
|  | *Selenomonas* | - | 0.82 | 7.74 | 3.4E-06 |
|  | *Fibrobacter* | - | -1.19 | 8.50 | 6.2E-04 |
|  | *Candidatus Methanomethylophilus* | - | -0.82 | 5.17 | 2.6E-03 |
|  | *Ruminococcus* | - | -0.59 | 7.58 | 3.3E-05 |
| Genes |  |  |  |  |  |
|  | *echB* | Central methanogenic pathway | 0.49 | 5.62 | 1.6E-04 |
|  | *fwdB* | Hydrogenotrophic methanogenesis | 0.46 | 5.51 | 2.6E-04 |
|  | *echA* | Central methanogenic pathway | 0.41 | 6.37 | 5.4E-04 |
|  | *fwdF* | Hydrogenotrophic methanogenesis | 0.49 | 5.67 | 1.5E-03 |
|  | *mtmB* | Methylotrophic methanogenesis | -1.40 | 5.05 | 1.0E-07 |
|  | *mtaB* | Methylotrophic methanogenesis | -0.87 | 5.29 | 4.4E-08 |
|  | *glpX* | Aerobic oxidation of methane | -0.60 | 7.51 | 5.5E-06 |
|  | *pqqF* | Aerobic oxidation of methane | -0.55 | 8.02 | 3.5E-05 |
|  | *fdhA-K00148* | Aerobic oxidation of methane | -0.55 | 7.94 | 2.0E-05 |
|  | *fpoJ* | Central methanogenic pathway | -0.53 | 5.50 | 4.7E-04 |
|  | *metF* | Hydrogenotrophic methanogenesis | -0.51 | 9.45 | 1.1E-04 |
|  | *fpoC* | Central methanogenic pathway | -0.50 | 5.61 | 6.2E-06 |
|  | *rnfD* | Central methanogenic pathway | -0.50 | 10.38 | 4.7E-05 |

**Supplementary Table S4. Number of reads per sample analyzed by 2 taxonomic profiling protocols (mean [min – max])**

| **Step** | **Standard protocol** | | |  | **Customized protocol** | | |
| --- | --- | --- | --- | --- | --- | --- | --- |
|  | **Sequencing reads per sample (x10^6^)** | **Proportion of initial sequencing reads (%)** | **Proportion of non-host reads (%)** |  | **Sequencing reads per sample (x10^6^)** | **Proportion of initial sequencing reads (%)** | **Proportion of non-host reads (%)** |
| Raw sequences | 54.2 [30.5 - 75.8] | 100 | - |  | 54.2 [30.5 - 75.8] | 100 | - |
| Sequence trimming | 52.3 [29.2 - 73.2] | 96.5 | - |  | 52.3 [29.2 - 73.2] | 96.5 | - |
| Host decontamination | 41.9 [24.9 - 59.9] | 77.3 | 100 |  | 38.3 [23.3 - 55.2] | 70.7 | 100 |
| Taxonomic classification |  |  |  |  |  |  |  |
| Confidence score = 0 | 5.9 [3.5 - 8.2] | 10.9 | 14.1 |  | 8 [4.9 - 11.3] | 14.8 | 21 |
| Confidence score = 0.1 | 1.3 [0.7 - 2] | 2.4 | 3.1 |  | 1.6 [0.9 - 2.3] | 3 | 4.2 |

**Supplementary Table S5. Read classification rates by taxonomic level.**

| **Taxonomic Rank** | **Number of rank-specific classified reads/non-host reads (%)** | **Number of rank-specific classified reads/total number of classified reads (%)** |
| --- | --- | --- |
| Domain | 4.15 | 100.0 |
| Kingdom | 3.99 | 96.2 |
| Phylum | 3.99 | 96.2 |
| Class | 3.95 | 95.1 |
| Order | 3.94 | 94.9 |
| Family | 3.87 | 93.2 |
| Genus | 3.77 | 91.0 |
| Species | 3.14 | 75.7 |

**Supplementary Figure 1. Results of A) weight collection and B-C) average daily weight (ADG) calculation.** Error bars in A represent standard deviation (SD), while in B and C boxes represent the 25th to 75th percentile, horizontal line represents the median and whiskers indicate 1.5× the interquartile range.

**Supplementary Figure 2. Relative abundance of genus-level microbiome composition for A) Positive and B) Negative controls.** Most abundant species are shown, and species < 1% abundance are grouped as “Others”. Each bar represents a single sample.

**Supplementary Figure 3. Relative abundance plot of genus-level bacterial (A) and archaeal (B) composition, grouped by weaning strategy and collection day**. Genera with < 1% abundance are grouped as “Others”. Each columns represents a single sample.


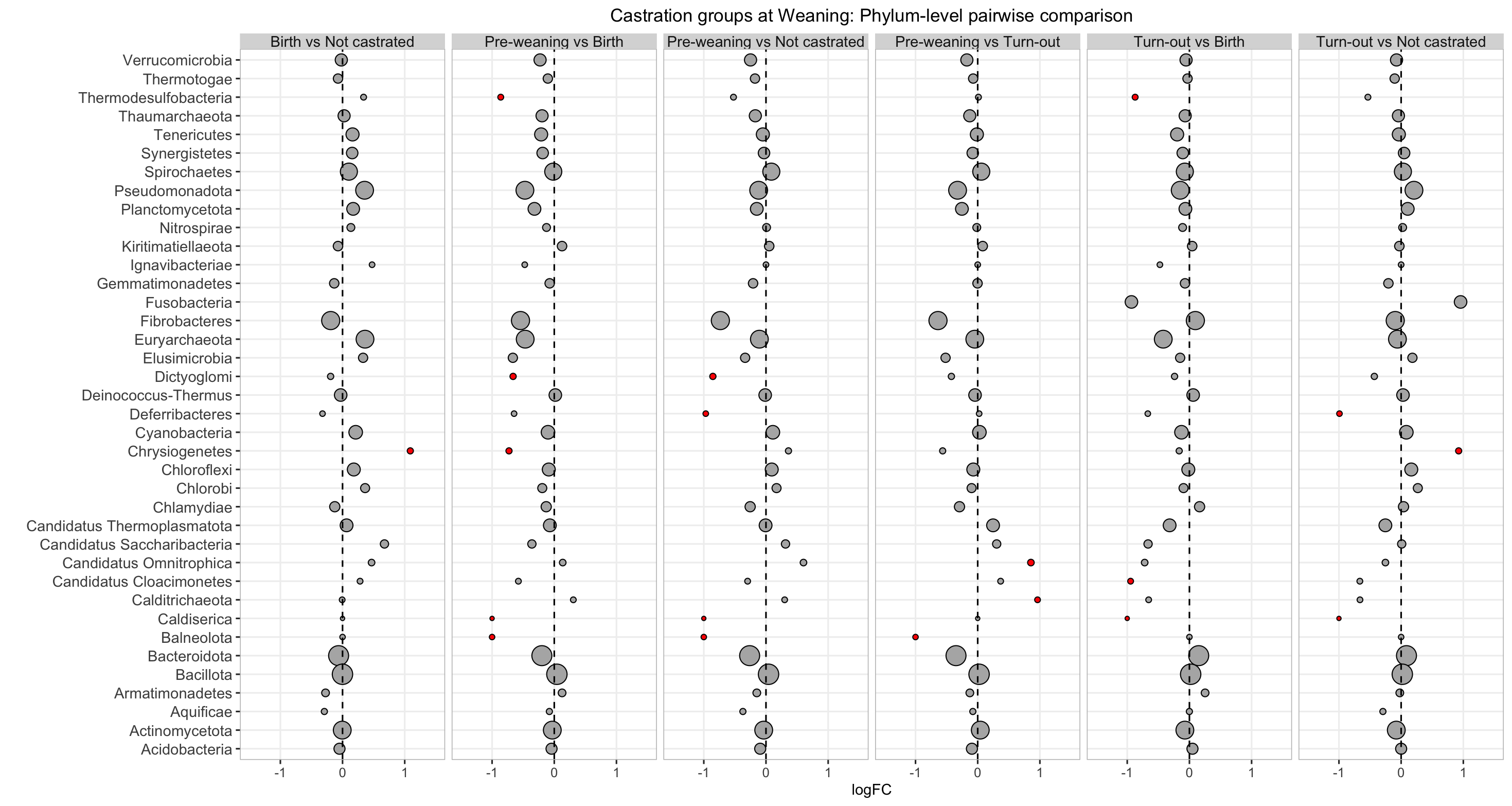


**Supplementary Figure 4. Differential abundance of microbial phyla between castration timing groups at weaning day expressed as log2 fold change (LogFC).** Statically significant logFC (adjusted *P*<0.05) are depicted in red and non-significant in grey. Circle diameter is proportional to the average abundance of each phylum across all samples.

**Supplementary figure 5. Methane-associated genera and gene content in the rumen, stratified by collection day and weaning strategy. A) Relative abundance plot of genus-level methane-associated microbe composition grouped by collection day and weaning strategy.** Taxa with < 1% relative abundance are grouped as “Others”. Each bar corresponds to an individual sample. **B) Alpha and C) Beta diversity plots at the methane-associated gene level.** Shannon Index is depicted as an alpha diversity index (Boxes represent the 25^th^ to 75^th^ percentile; horizontal line represents the median; and whiskers indicate 1.5× the interquartile range, p-values from Type-III ANOVA). Beta diversity is depicted in a Non-Metric Multidimensional Scaling (NMDS) ordination plot based on Bray–Curtis distances *(P* and R2 values from PERMANOVA testing).
